# Enhancing Radiologist Productivity with Artificial Intelligence in Magnetic Resonance Imaging (MRI): A Narrative Review

**DOI:** 10.3390/diagnostics15091146

**Published:** 2025-04-30

**Authors:** Arun Nair, Wilson Ong, Aric Lee, Naomi Wenxin Leow, Andrew Makmur, Yong Han Ting, You Jun Lee, Shao Jin Ong, Jonathan Jiong Hao Tan, Naresh Kumar, James Thomas Patrick Decourcy Hallinan

**Affiliations:** 1 Department of Diagnostic Imaging, National University Hospital, 5 Lower Kent Ridge Rd, Singapore 119074, Singapore; arun.nair@mohh.com.sg (A.N.); wilson.ong@mohh.com.sg (W.O.); aric.lee@mohh.com.sg (A.L.); andrew_makmur@nuhs.edu.sg (A.M.); yonghan_ting@nuhs.edu.sg (Y.H.T.); youjun.lee@mohh.com.sg (Y.J.L.); shao_jin_ong@nuhs.edu.sg (S.J.O.); 2 AIO Innovation Office, National University Health System, 3 Research Link, #02-04 Innovation 4.0, Singapore 117602, Singapore; naomi_wenxin_leow@nuhs.edu.sg; 3 Department of Diagnostic Radiology, Yong Loo Lin School of Medicine, National University of Singapore, 10 Medical Drive, Singapore 117597, Singapore; 4 National University Spine Institute, Department of Orthopaedic Surgery, National University Health System, 1E Lower Kent Ridge Road, Singapore 119228, Singapore; jonathan_jh_tan@nuhs.edu.sg (J.J.H.T.); dosksn@nus.edu.sg (N.K.)

**Keywords:** MRI, artificial intelligence, radiology workflow, productivity, deep learning, automated segmentation, radiologist efficiency, machine learning, worklist triage

## Abstract

Artificial intelligence (AI) shows promise in streamlining MRI workflows by reducing radiologists’ workload and improving diagnostic accuracy. Despite MRI’s extensive clinical use, systematic evaluation of AI-driven productivity gains in MRI remains limited. This review addresses that gap by synthesizing evidence on how AI can shorten scanning and reading times, optimize worklist triage, and automate segmentation. On 15 November 2024, we searched PubMed, EMBASE, MEDLINE, Web of Science, Google Scholar, and Cochrane Library for English-language studies published between 2000 and 15 November 2024, focusing on AI applications in MRI. Additional searches of grey literature were conducted. After screening for relevance and full-text review, 67 studies met inclusion criteria. Extracted data included study design, AI techniques, and productivity-related outcomes such as time savings and diagnostic accuracy. The included studies were categorized into five themes: reducing scan times, automating segmentation, optimizing workflow, decreasing reading times, and general time-saving or workload reduction. Convolutional neural networks (CNNs), especially architectures like ResNet and U-Net, were commonly used for tasks ranging from segmentation to automated reporting. A few studies also explored machine learning-based automation software and, more recently, large language models. Although most demonstrated gains in efficiency and accuracy, limited external validation and dataset heterogeneity could reduce broader adoption. AI applications in MRI offer potential to enhance radiologist productivity, mainly through accelerated scans, automated segmentation, and streamlined workflows. Further research, including prospective validation and standardized metrics, is needed to enable safe, efficient, and equitable deployment of AI tools in clinical MRI practice.

## 1. Introduction

In the last two decades, artificial intelligence (AI), particularly machine learning (ML) and natural language processing (NLP), have significantly transformed the field of radiology [1]. AI applications have demonstrated promise in reducing the diagnostic workload of radiologists while potentially enhancing productivity and diagnostic accuracy across various modalities. Magnetic resonance imaging (MRI) plays a crucial role in modern diagnostics due to its superior soft-tissue contrast, multi-planar capabilities, and lack of ionizing radiation, making it essential for diagnosing a wide range of conditions, from neurological disorders to musculoskeletal injuries [2]. As of 2022, there are an estimated 50,000 MRI systems installed worldwide, with over 95 million MRI scans performed yearly [3]. Despite the widespread use and critical importance of MRI in clinical diagnostics, there has been limited systematic assessment of how AI applications affect clinical productivity within MRI workflows across various specialties. Despite novel advancements in deep learning (DL) for computer imaging with increased emphasis on quality optimization whilst maintaining accuracy [4,5,6,7,8], it remains to be seen if these can be translated into medical imaging (specifically MRI) whilst optimizing time gains or losses.

The complexity of MRI interpretation significantly contributes to radiologists’ increased workload and the potential for diagnostic delays. Radiologists are tasked with analyzing large volumes of intricate images—often in the thousands—requiring the analysis of one image every 3–4 s during a typical 8 h workday to meet workload demands [9]. Furthermore, interobserver variability can result in inconsistent diagnoses, underscoring the need for tools that standardize and improve diagnostic accuracy [10]. AI holds promise in addressing these challenges by automating routine tasks, optimizing scan protocols, and aiding in image interpretation, ultimately enhancing workflow efficiency and reducing diagnostic errors [2,11].

Previous reviews have primarily focused on specific subspecialties or examined AI applications across multiple imaging modalities, often offering limited insight into the implications of AI specifically within MRI [12,13,14,15]. The heterogeneity in study designs, endpoints, and performance metrics makes it difficult to conduct a formal meta-analysis with robust reasoning [16]. Given the wide variations in study designs, AI models, clinical settings, and outcome measures, a narrative review is more suitable. This approach allows for the synthesis of findings and insights despite the lack of uniformity, providing a flexible framework to explore the impact of AI on MRI.

Measuring productivity in radiology is multifaceted. While time savings and increased throughput are often-cited benefits of AI, other aspects such as diagnostic accuracy, workflow efficiency, and reduction in radiologist fatigue are equally important [17,18]. However, there is no clear consensus on which metrics should be used to assess these outcomes. Studies often focus on specific applications of AI—such as lesion detection, scan protocolling and scan prioritization—which limits the ability to make an overall assessment of AI’s contribution to productivity [19,20,21]. Furthermore, AI models developed in specific institutions or datasets may not generalize well to broader clinical practice, raising concerns about bias and reproducibility [22,23]. Ethical considerations, including data privacy and the need for transparency in AI algorithms, also play a significant role in adoption of AI in MRI [24]. Radiologist acceptance of AI tools depends on their reliability and ability to integrate seamlessly into existing workflows, highlighting the importance of collaborative efforts between AI developers and clinicians [25].

This review aims to provide a comprehensive overview of AI applications in MRI across different clinical specialties, with a particular focus on their potential to enhance radiologist productivity during scanning and/or interpretation. We will examine studies that assess AI tools in terms of time savings, accuracy improvements, and workflow integration, while also identifying gaps in the literature where further research is needed. By addressing the current landscape and identifying areas for improvement, we hope to inform future research and guide the practical integration of AI tools in MRI.

## 2. Materials and Methods

### 2.1. Literature Search Strategy

In order to enhance the transparency and completeness of our review, we employed a systematic search with our narrative review methodology. Initial search strategy was performed according to Preferred Reporting Items for Systematic Reviews and Meta-Analyses (PRISMA) guidelines [26].

On 15 November 2024, a literature search of the major databases was conducted (PubMed, EMBASE, MEDLINE, Web of Science, Google Scholar, and Cochrane library) using the following strategy: (productivity OR productiveness OR prolificacy) AND (radiology OR radiologist) AND (artificial intelligence OR AI) AND (magnetic resonance imaging OR MRI). Limits were applied to only include English language studies published between the year 2000 and 15 November 2024.

Additional grey literature searches have also been conducted via hand search (OATD, OpenGrey, OCLC, NIH Clinical Trials, TRIP medical database).

### 2.2. Study Screening and Selection Criteria

A two-stage screening process was used. Studies were first screened by title and abstract (A.N). A full text review was then performed for any potentially eligible studies. Any controversies at either stage were reviewed by multiple authors (W.O., A.L, J.T.P.D.H) during assessment for study eligibility.

The inclusion criteria were as follows: studies focusing on the topic of using AI in MRI to enhance productivity, English studies, and studies performed on human subjects. The exclusion criteria were as follows: non-original research (for example, review articles and editorial correspondences), unpublished work, non-peer reviewed work, conference abstracts, and case reports. Studies focused primarily on other imaging modalities (for example radiographs, CT, or nuclear medical imaging) were also excluded.

### 2.3. Data Extraction and Reporting

The selected studies were extracted and compiled onto a spreadsheet using Microsoft Excel Version 16.81 (Microsoft Corporation, Washington, DC, USA). The following data were extracted:

1. Study details: title, authorship, year of publication and journal name.
2. Application and primary outcome measure.
3. Study details: sample size, region studied, MRI sequences used.
4. Artificial intelligence technique used.
5. Key results and conclusion.

## 3. Results

### 3.1. Search Results

**Figure 1:**
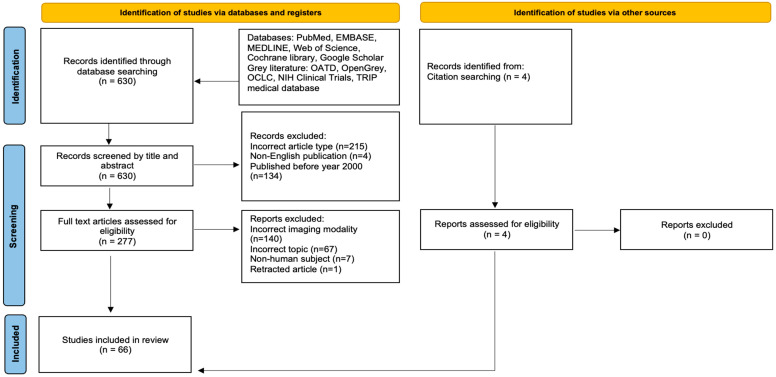
PRISMA flowchart showing the two-step study screening process. Adapted from PRISMA Group, 2020 [26].

Our initial literature search identified 630 studies following screening to the specified criteria and removal of any duplicates. Subsequently, 134 studies which did not meet the date range, 215 of incorrect article types (as specified in the exclusion criteria), and 4 non-English publications were initially excluded during screening by title and abstract alone. This led to 277 studies being selected for full screening. Four additional studies were also identified from citation searching and assessed for eligibility during the full screening. The end result was the inclusion of 66 studies in the present review (see Figure 1 for a detailed flowchart). The articles were summarized based on data extracted in Table 1 and Table 2.

We classified the included studies based on the following themes: Reducing scan times to improve efficiency (13/66, 19.7%), automating segmentation (26/66, 39.4%), optimizing worklist triage and workflow processes (7/66, 10.6%), decreasing reading times (16/66, 24.2%), and time-saving and workload reduction (6/66, 9.1%). In terms of overall productivity gains, around half of the studies (35/66, 53.1%) demonstrated this clearly across one or more of the themes identified. The remaining studies were deemed to have unclear productivity gains based on the limitations highlighted in Table 2 and subsequent discussion, with a few productivity losses (3/66, 4.5%) due to the use of AI also seen.

### 3.2. Overview of How Can AI Help in MRI Interpretation

**Figure 2:**
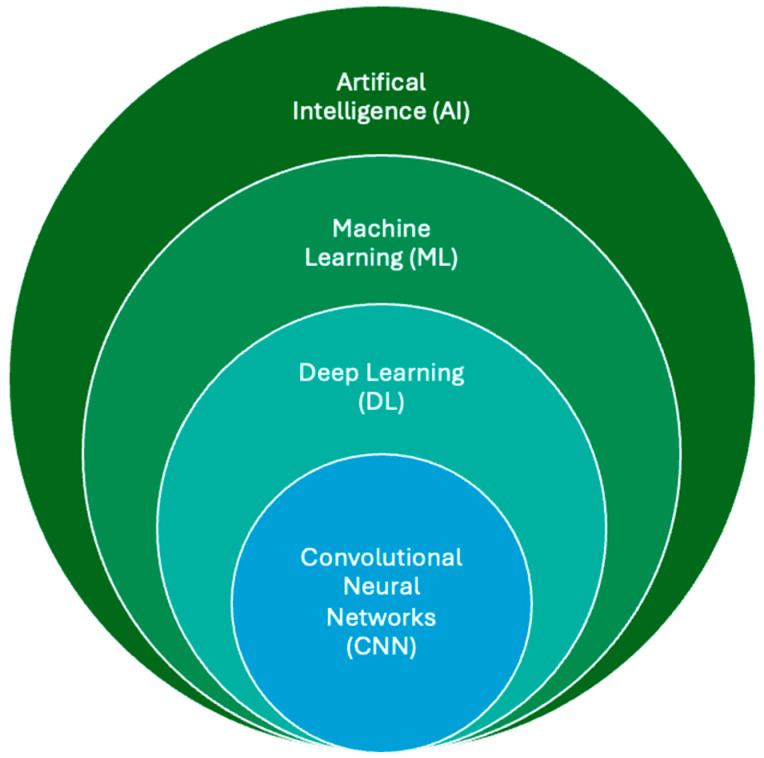
The relationship between AI, ML, DL, and CNN. ML lies within the field of AI and allows computer systems to learn without explicit programming or knowledge. As a subsection of machine learning, deep learning uses computational models similar to the neuronal architecture within the brain, simulating multilayer neural networks in order to resolve complex tasks. A CNN automatically learns and adapts to spatial hierarchies of features through backpropagation using multiple building blocks, allowing analysis of 2D and 3D medical images.

Artificial intelligence (AI) refers to the use of computers and technology to perform tasks that mimic human intelligence and critical thinking [91]. First introduced at the 1956 Dartmouth Conference, AI gained significant traction in medicine during the third AI boom in the 2010s, driven by advancements in machine learning (ML) and deep learning (DL) [92]. ML involves training models to make predictions using existing datasets, while DL employs artificial neural networks modeled on the brain’s architecture to solve complex problems [93,94]. The exponential growth in digital imaging and reporting data have further accelerated advancements in medical imaging, particularly through the application of convolutional neural networks (CNNs), a key subset of DL. CNNs have revolutionized medical imaging by enabling the development of computer-aided diagnostic (CAD) systems, which assist radiologists in detecting lesions and tumors across various imaging modalities, including MRI [95]. These systems aim to enhance diagnostic accuracy and efficiency, reducing workload and interobserver variability. Many CAD systems rely on CNN models to deliver expert-level performance across different areas of imaging research [96]. The relationship between AI, ML, DL, and CNN is depicted in Figure 2, highlighting the interconnected nature of these technologies and their role in advancing medical imaging.

**Figure 3:**
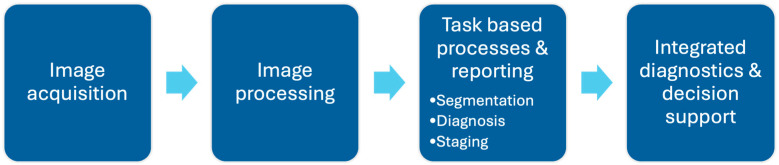
Outline of AI application in radiology workflow in typical clinical setting. AI has potential in reducing scan times during image acquisition and processing, support specific image-based task processes such as segmentation/diagnosis/staging, reduce reading time during reporting, and improve integrated diagnostic processes beyond the reporting phase, including deciding appropriate treatment plans and evaluating prognosis in patients.

While AI has demonstrated success in improving accuracy, enhancing efficiency is equally critical to facilitate the seamless integration of these models into clinical practice [97]. The clinical application of AI in MRI can be broadly categorized into five key steps: image acquisition, image processing, task-specific applications, reporting, and integrated diagnostics. This workflow is further illustrated in Figure 3.

### 3.3. Reducing Scan Times to Improve Efficiency

Implementing more efficient protocols and eliminating unnecessary sequences can significantly reduce scan times, streamlining the initial stages of image acquisition and processing. This, in turn, eases the pressure on subsequent steps, such as reducing MRI reading times, ultimately helping to alleviate radiologist fatigue.

One key example is the improvements in AI-driven lung morphometry using diffusion-weighted MRI (DW-MRI). A deep cascade of residual dense networks (DC-RDN) has enabled the rapid production of high-quality images [32]. This approach reduced the required breath-holding time from 17.8 s to 4.7 s, with each slice (64 × 64 × 5) reconstructed within 7.2 ms.

In one study [88] exploring an MRI acceleration framework to reconstruct dynamic 2-D cardiac MRI sequences from undersampled data, a deep cascade of convolutional neural networks (CNNs) was successfully used to accelerate data acquisition. This approach resulted in improvements in both image quality and reconstruction speed. Each complete dynamic sequence was reconstructed in under 10 s, with each 2-D image frame taking only 23 ms. Faster reconstruction enables more timely image availability for diagnosis and intervention, directly contributing to increased radiologist throughput.

For assessing hepatobiliary phase (HBP) adequacy in gadoxetate disodium (EOB)-enhanced MRI, a convolutional neural network (CNN) was shown to evaluate HBP adequacy in real time, potentially reducing examination times by up to 48% for certain patient groups [40]. In gastric cancer workups, a deep learning-accelerated single-shot breath-hold (DSLB) T2-weighted imaging technique demonstrated superior quality, faster acquisition times, and improved staging accuracy compared to current BLADE T2-weighted imaging methods [46]. DSLB reduced the acquisition time of T2-weighted imaging from a mean of 4 min 42 s per patient to just 18 s per patient while delivering significantly better image quality, with 9.43-fold, 8.00-fold, and 18.31-fold improvements in image quality scores across three readers compared to the standard BLADE.

In spinal MRI, one study [56] utilized a deep learning-based reconstruction algorithm combined with an accelerated protocol for lumbar MRI, reducing the average acquisition time by 32.3% compared to standard protocols while maintaining image quality. A separate study [55] on lumbar central canal stenosis (LCCS) demonstrated improved measurement accuracy using sagittal lumbar MR slices alone, compared to models that combined sagittal and axial slices. This approach showed robustness across moderate-to-severe LCCS cases, suggesting that automated LCCS assessment using sagittal T2 MRI alone could reduce the need for additional axial imaging, thereby shortening scan times.

However, limitations remain. Prior studies on DL techniques for MRI acceleration [98,99] did not specify improvements across all full spine MRI examination sequences, and their clinical utility was constrained by the lack of multicenter validation. Despite these caveats, DL-based reconstruction in musculoskeletal (MSK) MRI offers a promising direction for reducing examination times. For instance, one study [100] demonstrated that a 10 min accelerated 3D SPACE MRI protocol for the knee delivered comparable image quality and diagnostic performance to a 20 min 2D TSE MRI protocol.

A novel study [89] on MRI of the knee introduced a variational network framework that integrates data consistency with deep learning-based regularization, enabling the reconstruction of high-quality images from undersampled MRI data. This method outperformed non-AI reconstruction algorithms by a factor of 4 and achieved reconstruction times under 193 ms on a single graphics card, requiring no parameter tuning once trained. These characteristics make it easily integrable into clinical workflows.

DL models [19,66,67,68] have also been developed to accelerate reconstruction times for various brain MRI sequences while maintaining accuracy and improving image quality. These models enhance the signal-to-noise ratio (SNR) and reduce artifacts, such as those caused by head motion. In one study [69], an accelerated DL reconstruction method improved image quality and reduced sequence scan times by 60% for brain MRI.

Not all studies demonstrated reduced scan times despite improvements in image quality. For instance, a DL-based reconstruction model for intracranial magnetic resonance angiography (MRA) significantly improved SNR and contrast-to-noise ratio (CNR) for basilar artery analysis [76], but increased scan times, potentially affecting clinical productivity. Similarly, for shoulder MRI, while DL-based reconstructions significantly improved SNR and CNR, they showed inconsistencies in reducing scan times when applied [60,61].

### 3.4. Automating Segmentation

A significant majority of the included studies focused on automating segmentation in MRI across various specialties. Manual segmentation is often time-consuming and labor-intensive for radiologists [50]. AI models provide a promising solution by easing this workload and enabling radiologists to deliver more concise and time-efficient imaging-based diagnoses.

Multiple studies [27,28,29] focusing on breast tumor have demonstrated the successful development of AI programs for automating tumor segmentation in dynamic contrast-enhanced MRI (DCE-MRI). One such program achieved a 20-fold reduction in manual annotation time for expert radiologists, while another showed the potential to reduce unnecessary biopsies of benign breast lesions by up to 36.2%. Automated tumor segmentation not only enhances productivity but also minimizes interobserver variability, leading to more consistent and accurate diagnoses.

For lung upper airway segmentation in static and dynamic 2D and 3D MRI, a study utilizing the VGG19 architecture [33] demonstrated a deep learning segmentation system that was statistically indistinguishable from manual segmentations, accounting for human variability. This represents a significant improvement over earlier models using classic augmentation, enabling more efficient structure identification. However, time savings were not detailed, leaving questions about overall efficiency in broader clinical applications.

In head, neck, and lung oncology, AI-based approaches to radiation planning have shown significant time savings with automated segmentation models combing various imaging modalities, including MRI [90,101]. These models eliminated labor-intensive, observer-dependent manual delineation of organs-at-risk (OARs). One study reported average model runtimes of 56 s and 3.8 min, compared to 12.4 min for clinical target volume (CTV) delineation and 30.7 min for OAR delineation by junior radiation oncologists. These time reductions allow radiologists and radiation oncologists to focus on complex decision-making tasks and patient care.

Studies on hepatocellular carcinoma detection and liver segmentation [34,35,36,102] have shown that radiomic and deep learning approaches can automate segmentation and delineation of pathological subtypes across MRI sequences, achieving 10- to 20-fold improvements in measurement time compared to manual methods.

AI pipelines for MRI have also demonstrated potential in automating segmentation and reducing contouring time for renal lesions [47,48]. In one study, combining MRI and AI for renal lesion differentiation during follow-up resulted in cost savings over 10 years, with total estimated costs of $8054 for the MRI strategy and $7939 for the MRI + AI strategy. This approach also yielded marginal gains in quality-adjusted life years (QALYs), demonstrating potential for reallocating radiologist resources to other tasks. Additionally, a novel automated deep learning-based abdominal multiorgan segmentation (ALAMO) technique [49] successfully segmented multiple OARs for MR-only radiation therapy and MR-guided adaptive radiotherapy. This technique offers potential time savings and reduces fatigue by replacing manual delineation with comparable accuracy.

For shoulder pathologies such as rotator cuff tears, deep learning frameworks utilizing parameters like occupation ratio and fatty infiltration [58] have achieved fast and accurate segmentation of the supraspinatus muscle and fossa from shoulder MRI. These frameworks enhance diagnostic efficiency and objectivity, with some performance metrics surpassing those of experienced radiologists [59].

In brain MRI segmentation, skull stripping is a critical preprocessing step that removes non-brain tissue (e.g., skull, skin, and eyeballs) to reduce computational burden and misclassification risks, enabling more targeted analysis of brain tissue [103]. One deep learning model using anatomical context-encoding network (ACEnet) [64] could segment an MRI head scan of 256 × 256 × 256 within approximately 9 s on a NVIDIA TITAN XP GPU, facilitating potential real-time applications. The model achieved Dice scores greater than 0.976. Another application [65] focused on cerebrospinal fluid (CSF) volume segmentation, demonstrating strong reliability in measuring age-related brain atrophy. This application automatically segmented brain and intracranial CSF and measured their volumes and volume ratios within 1 min, using a popular 3D workstation in Japan. For brain pathologies, a CNN model [70] accurately segmented meningiomas and provided volumetric assessments during serial follow-ups, achieving a median performance of 88.2% (within the inter-expert variability range of 82.6–91.6%). The study also reported a 99% reduction in segmentation time, averaging 2 s per segmentation (*p* < 0.001).

Automated segmentation has also enabled assessments of generalized parameters, such as tissue volume and distribution. One deep learning pipeline [62] automated measurements across various landmarks in the lower limbs. While this study reported time gains rather than savings, the potential for improved clinical value in diagnosing or monitoring lipoedema or lymphedema represents a potential productivity enhancement. For rarer hand tumors, a CNN-based segmentation system [63] improved tumor analysis on hand MRIs, though its accuracy (71.6%) was limited by a small sample size and narrow tumor selection.

Advances in image segmentation have extended to niche subspecialties in MRI. In neonatology, models integrating clinical variables (e.g., gestational age and sex) with automated segmentation techniques [77,78,79] have been used to screen for pathologies such as hypoxic–ischemic encephalopathy (HIE) and predict neurodevelopmental outcomes up to two years later. In nasopharyngeal carcinoma (NPC) treatment, a deep learning-based segmentation model [80] accurately delineated gross tumor volume (GTV), surpassing previous methods and improving clinicians’ efficiency in creating radiation plans for targeted therapies.

AI models have also been applied to sleep apnea evaluation. A study [22] using deep learning-based segmentation of the pharynx, tongue, and soft palate on mid-sagittal MRI efficiently identified sleep apnea-related structures. Results were consistent with intra-observer variability, and the model’s processing time of only 2 s on a modern GPU demonstrates its potential for real-time clinical applications, such as computer-aided reporting.

### 3.5. Optimizing Worklist Triage and Workflow Processes

A multicenter study [30] developed an automated sorting tool for cardiac MRI examinations by linking examination times with appropriate post-processing tools. This tool directed images that did not require quantitative post-processing to PACS more quickly for interpretation. By streamlining the workflow, radiologists received cardiac MRI images sooner, enhancing clinical efficiency. However, the tool demonstrated poor accuracy when handling images with high variability in acquisition parameters, such as perfusion imaging across different centers, highlighting a key limitation.

AI has also made significant progress in prostate MRI, particularly in the early detection and classification of prostate cancer [45]. One proposed model [42] aimed to optimize worklist triage by automating the grading of extra-prostatic extension using T2-weighted MRI, ADC maps, and high b-value DWI sequences. This approach shows promise for improving workflow efficiency in prostate cancer evaluations.

In the workup for hepatocellular carcinoma (HCC), an AI model demonstrated the ability to differentiate between pathologically proven HCC and non-HCC lesions, including those with atypical imaging features on MRI [36]. This CNN-based model achieved an overall accuracy of 87.3% and a computational time of less than 3 ms, suggesting its potential as a clinical support tool for prioritizing worklists and streamlining MRI workflows.

Workflow efficiencies can also be achieved through improved and automated protocoling of MRI studies. A recent study [104] using a secure institutional large language model demonstrated significantly better clinical detail in spine MRI request forms and an accurate protocol suggestion rate of 78.4%. Another study [86] on musculoskeletal MRI evaluated a CNN-based chatbot-friendly short-text classifier, which achieved 94.2% accuracy. The study suggested extending this text-based framework to other repetitive radiologic tasks beyond image interpretation to enhance overall efficiency.

For breast malignancy evaluation using MRI, artificial intelligence has shown great potential in improving diagnostic accuracy and workflow efficiency. A notable multicenter study [87] utilized machine learning algorithms to identify axillary-tumor radiomic signatures in patients with invasive breast cancer. This approach successfully detected axillary lymph node metastases at early stages, which is crucial for surgical decision-making. Integrating such AI tools into clinical workflows could help radiologists prioritize scans more effectively and reduce the need for additional surgical interventions in select patients.

Beyond assisting with report generation, several studies have targeted workflow improvements in integrated diagnostics, including decision support and outcome prediction. While these advancements currently have limited impact on routine reporting practices, the growing demand for real-time data and follow-up reporting could significantly burden radiologists if workflow efficiencies are not implemented [105].

Various AI models [37,38] have also demonstrated success in predicting mortality outcomes and early recurrence pre- and post-surgery. These models identified deep learning signatures linked to risk factors such as increased microvascular invasion and tumor number, outperforming conventional clinical nomograms in accuracy. However, most studies did not discuss how these innovations could be seamlessly integrated into clinical practice.

One AI model [39] focusing on hepatocellular carcinoma (HCC) described an automated risk assessment framework with a runtime of just over one minute per patient (0.7 s for automated liver segmentation, 65.4 s for extraction of radiomic features, and 0.42 s for model prediction). If implemented, such frameworks could enhance clinical reporting while maintaining workflow efficiency.

### 3.6. Decreasing Reading Times

Reading times can vary significantly depending on procedure type, individual radiologist, and specialty [106]. However, reducing reading time is a straightforward way to measure increased efficiency achieved by radiologists in a typical clinical workflow, assuming workload and report accuracy remain broadly consistent.

The use of AI has advanced to improve reporting times for post-processed images across various specialties. One CNN-based model [31], used for aortic root and valve measurements, achieved an average speed improvement of 100 times compared to expert cardiologists in the UK during image interpretation.

In spinal MR imaging, AI has brought multifaceted improvements, particularly in tasks where measurements by reporting radiologists are repetitive and time-consuming. One study [50] introduced a deep learning model for detecting and classifying lumbar spine central canal and lateral recess stenosis, achieving accuracy comparable to a panel of expert subspecialized radiologists. A subsequent study [51] focusing on productivity metrics for this model demonstrated significant reductions in reporting times. Similar performance metrics have been observed in other DL models [52,53,54] across various centers using different training datasets, such as SpineNet and Deep Spine. However, a key limitation is that time savings, if any, were not always clearly specified.

In musculoskeletal (MSK) imaging, AI applications have also reduced reading times, particularly for detecting ligament and meniscal injuries in the knee. A multicenter study [57] evaluating an AI assistant program reported marked improvements in accuracy (over 96%) and time savings in diagnosing anterior cruciate ligament (ACL) ruptures on MRI, particularly among trainee radiologists. Diagnostic time for trainees improved to 10.6 s (from 13.8 s) for routine tasks and 13.0 s (from 37.5 s) for difficult tasks, compared to 8.5 s (from 10.5 s) and 13.6 s (from 23.3 s), respectively, for expert radiologists.

In neuroradiological reporting of multiple sclerosis (MS) lesions, prior deep learning methods were limited due to error susceptibility. A multicenter study [85] introduced a novel automation framework called “Jazz”, which identified two to three times more new lesions than standard clinical reports. Reading time using “Jazz” averaged 2 min and 33 s, though the study did not compare this to the time required for a standard radiologist-generated report without AI assistance. Another multicenter study [72] tested a deep learning model to automatically differentiate active from inactive MS lesions using non-contrast FLAIR MRI images. The model achieved an average accuracy of up to 85%, but a significant limitation was its reliance on manually selected regions of interest (ROIs) already diagnosed and categorized by radiologists, thereby restricting productivity gains in decreased reading time. In other neurological presentations, various AI models [73,74,75] have demonstrated improvements in MRI related to Parkinson’s disease, with fully automated networks achieving accuracies of up to 93.5% and processing times of under one minute to distinguish between patients with Parkinson’s disease and healthy controls.

For prostate MRI scans, a DL-based computer-aided diagnosis (CAD) system [81] improved diagnostic accuracy for suspicious lesions by 2.9–4.4% and reduced average reading time by 21%, from 103 to 81 s. However, a study [82] examining an AI clinical decision support tool for brain MRI found significant improvements in performance among non-specialists in diagnosis, producing top differential diagnoses, and identifying rare diseases, while specialist performance showed no significant differences with or without the AI tool. This underscores the need to benchmark AI models against expert performance to justify their integration into radiologists’ workflows.

In prostate multiparametric MRI (mpMRI), a retrospective multicenter study [43] reported a significant reduction in radiologist reading time (by 351 s) when grading was AI-assisted. Another study [41] on prostate segmentation showed that, for prostate cancer diagnosis, AI-based CAD methods improved consistency in internal (κ = 1.000; 0.830) and external (κ = 0.958; 0.713) tests compared to radiologists (κ = 0.747; 0.600). The AI-first read (8.54 s/7.66 s) was faster than both the readers (92.72 s/89.54 s) and the concurrent-read method (29.15 s/28.92 s). Nonetheless, there is criticism that the training datasets currently used to validate deep learning CAD models for prostate cancer workup are inadequate. Insufficient training data have been shown to result in worse performance compared to expert panels of radiologists [44].

### 3.7. Time-Saving and Workload Reduction

Several studies have explored radiologist time-saving and workload reduction strategies that extend beyond previously discussed themes.

A deep learning model [20] utilizing time-resolved angiography with stochastic trajectories (TWIST) sequences was developed to identify normal ultrafast breast MRI examinations and exclude them from the radiologist’s workload when integrated into the workflow. The trained model achieved an AUC of 0.81, reducing workload by 15.7% and scan times by 16.6% through elimination of unnecessary additional sequences. This demonstrates AI’s potential to streamline imaging protocols and enhance workflow efficiency.

To address challenges in obtaining high-quality training data for AI models, one study [83] evaluated manual prostate MRI segmentation performed by radiology residents. The study found that residents achieved segmentation accuracy comparable to experts, suggesting that residents could be employed to create training datasets. This approach could alleviate work pressures on expert radiologists while maintaining the high-quality data necessary for AI development.

Exploring broader applications, a CNN [71] addressed limitations in learning from small or suboptimal MRI datasets using a progressive growing generative adversarial network augmentation model. By generating realistic MRIs of brain tumors, the model enabled accurate classification of gliomas, meningiomas, and pituitary tumors, achieving an accuracy of 98.54%. This augmentation model offers a novel method to reduce the burden on radiologists by minimizing the need to manually generate large training datasets for deep learning frameworks.

In spine MRI, a study [84] investigated the effectiveness of AI-generated reports. The study reported improvements in patient-centered summaries and workflow efficiency by assisting radiologists in producing concise reports. However, the technology also presented challenges, with 1.12% artificial hallucinations and 7.4% potentially harmful translations identified. These error rates highlight that, without optimization, the current implementation of such technology could increase radiologists’ workload, as they would need to verify AI-generated reports before sharing them with patients.

## 4. Discussion

This review highlights the wide-ranging AI solutions applied to MRI across multiple specialties. In particular, many studies focused on accelerating scan times, automating segmentation, optimizing workflows (e.g., MRI form vetting), and reducing radiologist reading times, with each contributing to improved productivity in different ways. In the following sections, we examine these findings in greater detail, beginning with a discussion of the dominant AI architectures employed. We then address the strengths and limitations of the current evidence, delve into the ethical and regulatory framework which is essential for real-world adoption, and conclude with future directions and recommendations that emphasize broader validation, feedback loops, and next-generation AI modalities.

### 4.1. Dominant AI Architectures and Approaches

In our review, the studies employed a range of AI-based techniques, with convolutional neural network (CNN) architectures being the most frequently utilized. CNNs can be customized [95] for a wide array of tasks, including image processing, lesion detection, and characterization. Notable architectures used in the models include ResNet, DenseNet, and EfficientNet, which address specific limitations of earlier CNN models. ResNet, for instance, mitigates the vanishing gradient issue, while EfficientNet reduces computational and memory loads, leading to enhanced performance in both accuracy and efficiency [20,47,48,54,72,107]. Additionally, U-Net, a widely adopted CNN-based architecture, enables rapid and precise image segmentation and is commonly integrated into studies focused on automating segmentation tasks in MRI.

Beyond CNNs, other AI-based techniques, including some emerging technologies, were also implemented. For example, certain models utilized machine learning-driven intelligent automation software [85]. Classical machine learning (ML) methods continue to excel in simpler feature extraction tasks and often demonstrate more consistent performance across certain imaging modalities [108]. However, DL-based neural networks, particularly CNNs, have been shown to outperform classical ML approaches in most MRI-related applications [109]. A key advancement driving the success of DL is the integration of self-supervised learning (SSL).

Unlike traditional supervised learning, which relies on manually labeled datasets for “ground truth” supervisory signals, SSL enables models to generate implicit labels from unstructured data. By leveraging pretext tasks and downstream tasks, SSL-based ML models can effectively analyze large volumes of unlabeled medical imaging data while reducing the need for extensive human fine-tuning [110]. This approach has facilitated previously impractical developments, such as automated anatomical tracking in medical imaging [111]. Beyond deep learning-based applications, SSL has also been incorporated into novel ML frameworks for MRI, including dynamic fetal MRI, functional MRI for autism and dementia diagnosis, and cardiac MRI, all of which have demonstrated promising results [112,113,114]. Although SSL may not directly enhance daily clinical workflow efficiency or reduce workload, it has the potential to accelerate the development of novel ML imaging techniques aimed at improving these aspects.

Another significant development since 2017 is the adoption of Transformer-based architectures for imaging tasks. Originally designed for sequence-to-sequence prediction, the Transformer architecture employs a self-attention mechanism that dynamically adjusts to input content, offering notable advantages over traditional CNNs in image processing tasks [115]. This innovation has also contributed to the emergence of large language models (LLMs), such as OpenAI’s Generative Pre-Trained Transformer (GPT), which leverage Transformer architecture and extensive text-based corpora to perform a wide range of natural language processing (NLP) tasks. Other foundational LLMs, including Bidirectional Encoder Representations from Transformers (BERT), have served as precursors to domain-specific models like Med-PaLM, which focuses on healthcare applications [116]. Unlike earlier ML architectures that relied on either SSL or unsupervised learning, LLMs primarily utilize SSL to derive labels from input data.

While LLMs have gained traction in various fields, including medical chatbot development [117], their application in MRI remains limited. Among the reviewed studies, only one implemented an LLM-based approach for MRI analysis [84], highlighting the need for further exploration in this domain. Future research could investigate the potential of LLMs to automate text-intensive radiology tasks, such as report generation and clinical protocol, thereby enhancing efficiency in radiological workflows.

### 4.2. Strengths and Limitations of Current Evidence

Some studies included in this review were sourced outside the initial search strategy to allow a more comprehensive discussion of certain topics. Because the journal archives and grey literature sources were limited to those commonly available in public or academic settings, the search was not exhaustive. Consequently, AI applications under vendor development or within non-commercial settings may have been overlooked. In addition, by adopting a broad scope that examines AI developments in MRI across multiple specialties, this review may have sacrificed depth in favor of breadth compared to more narrowly focused analyses.

The heterogeneity observed in the included research limited the feasibility of conducting a formal meta-analysis, thereby reducing the ability to draw robust, statistically aggregated conclusions. Variations in study design, patient population data, and MRI protocols (across either training and testing datasets) in turn reduce the reproducibility of the models discussed. Through issues such as data leakage, the same results are prevented from being obtained under conditions defined by other clinical settings [118]. With that said, the majority of studies (52/66, 78.8%) described attempts at internal cross-validation using different splits of training and test datasets. This would have helped prevent overfitting, thereby assisting in more reliable model generalization [119]. Apart from discrepancies in data use or preprocessing, computational costs and hardware variations are major sources of irreproducibility. Our included studies either did not discuss this information, or failed to simulate results using lower performance hardware or software. This posed a challenge in making direct comparisons between them, or deriving relevance for clinical practice.

A lack of external validation characterized the majority of included studies (48/66, 72.7%). This is not an uncommon limitation, with a 2019 meta-analysis [120] reporting that only 6% of 516 studies on novel AI applications in medical imaging included external validation as a key component. This is despite the fact that such validation is a definitive step to ensuring that models can generalize to diverse clinical settings, patient populations, and imaging protocols. In addition, aside from one study [48], the long-term impact and cost-effectiveness of AI applications were not thoroughly compared across different specialties.

Finally, the retrospective nature of many studies raises questions about their applicability to real-world clinical settings [121]. The potential sources of bias, including differences in patient demographics and potential conflicts of interest, were also not discussed in detail, which raises questions about the fairness and equity of certain AI solutions. Although this review sought to highlight productivity metrics enhanced by AI, most studies reported outcomes related primarily to time savings and diagnostic accuracy. Radiologist-specific productivity indicators, including reductions in repetitive tasks or improved workflow satisfaction, were often relegated to secondary outcomes, complicating cross-study comparisons.

### 4.3. Recommended Enhancements

#### 4.3.1. Regulatory Frameworks and Compliance

Regulatory and compliance factors are crucial for a successful and consistent translation of AI tools into routine MRI practice. Concerns related to patient privacy, data security, and algorithmic transparency must be balanced with the potential productivity gains [122]. Regulatory guidelines established by organizations such as the United States Food and Drug Administration (FDA) and CE marking often mandate rigorous validation of AI algorithms, including prospective trials and post-market surveillance [123,124]. However, significant heterogeneity persists among vendors regarding the deployment, pricing, and maintenance of these AI products for specific clinical applications [125]. This variability poses challenges for stakeholders in selecting the most appropriate AI solution.

To address these complexities, several radiological organizations, including the American College of Radiology (ACR), Canadian Association of Radiologists (CAR), European Society of Radiology (ESR), Royal Australian and New Zealand College of Radiologists (RANZCR), and Radiological Society of North America (RSNA), have released joint guidelines to support the development, implementation, and use of AI tools [126]. These guidelines prioritize patient safety, ethical considerations, seamless integration with existing clinical workflows, and the stability of AI models through ongoing monitoring. Nevertheless, as these guidelines do not directly govern AI products or vendor selection, the responsibility falls on individual radiology departments or institutions to establish tailored frameworks. These frameworks must enable the selection and integration of suitable AI solutions while adhering to region-specific data standards [127].

#### 4.3.2. Generalizability and Multi-Center Validation

To ensure fairness and equity in AI-driven MRI applications, future research should prioritize minimizing biases related to demographics and disease prevalence. For smaller institutions that may lack the resources to compile large datasets, multicenter collaboration and external validation are crucial. Although many AI models achieve high accuracy within their home institutions, differences in imaging protocols, sequences, and MRI hardware often remain underexplored, leading to model underspecification and limited generalizability [128,129]. By incorporating multicenter validation, models can be trained on larger, more diverse datasets from multiple regions, enabling the development of algorithms that adapt to varying clinical populations and imaging protocols. In cases where legislative constraints hinder multicenter data sharing, synthetic data augmentation offers an alternative strategy to enhance dataset diversity. Publicly available, anonymized datasets, such as PROSTATEx, have already been utilized in some of the reviewed studies [70,81] and were recently combined with regional datasets to facilitate a novel multicenter, multi-scanner validation study for multiparametric MRI in prostate cancer [130].

Another approach to improving generalizability is the adoption of a federated learning (FL) framework, which has gained increasing recognition in medical AI research [131]. FL enables institutions to collaboratively develop machine learning models while maintaining data privacy, as each institution retains local control over its dataset and model updates are orchestrated through a centralized server. This framework allows institutions to selectively share or receive data within a trusted execution environment. However, FL presents challenges, including concerns about data heterogeneity and the protection of sensitive medical information [132]. Additionally, stress testing is an effective strategy to address model underspecification and improve robustness. By simulating variations in imaging parameters, introducing artificial noise, and modifying datasets to mimic real-world conditions, stress testing can identify model vulnerabilities. While studies on stress testing in MRI-based AI remain limited, findings from other imaging domains suggest its potential benefits [133].

From an implementation perspective, several infrastructure-related challenges must be addressed to enable real-world AI deployment in MRI. Many hospitals lack in-house machine learning expertise, necessitating collaborations with external research groups or commercial organizations. These partnerships often rely on third-party cloud-based computing systems, raising concerns regarding data security and privacy [134]. Furthermore, seamless integration of AI tools into existing radiology information systems (RIS) and picture archiving and communication systems (PACS) is essential for widespread adoption. Given the complexity and variability of radiology IT infrastructures, the development of open-source, vendor-agnostic PACS-AI platforms holds promise in addressing these integration challenges [135,136].

Beyond technical considerations, adequate human IT support and radiologist training are critical to ensuring the successful implementation of AI in clinical practice. Without appropriate infrastructure and user engagement, even the most advanced AI models may struggle to demonstrate tangible benefits in real-world settings. A recent systematic review [137] highlighted a significant gap in AI education among radiologists and radiographers, despite widespread enthusiasm for its adoption. This challenge is further exacerbated by the “black box” nature of certain AI models, which obscure decision-making processes and hinder practitioners’ ability to validate outputs [138]. Moreover, standardized training programs for imaging professionals in AI applications remain largely absent [139]. Increasing support for “white box” AI systems, which provide greater interpretability, alongside the development of dedicated educational platforms, could enhance transparency and trust. By equipping healthcare professionals with the knowledge to assess AI-generated recommendations and communicate their implications effectively, these efforts could ultimately foster greater public confidence in AI-driven medical technologies.

#### 4.3.3. Ethical Considerations

The expansion of digital healthcare has heightened concerns regarding ethical data usage and patient confidentiality. As hospitals and imaging centers rapidly upgrade IT systems to integrate AI tools, cybersecurity measures sometimes fail to keep pace. This has led to well-documented breaches of medical record databases and instances of fraudulent claims, exacerbating patient mistrust in AI-driven healthcare solutions [122]. In response, some regions have incorporated ethical AI guidelines into their legal frameworks [140]. For instance, the European Parliament’s AI Act, adopted in 2024 and set for full enforcement by 2026, complements existing legislation such as the General Data Protection Regulation (GDPR) and the Data Act. The AI Act establishes requirements for safety, reliability, and individual rights while fostering innovation and competitiveness. As the continued expansion of AI in healthcare relies on access to large-scale patient datasets, implementing robust regulatory frameworks is essential to maintaining public trust.

Hospitals and imaging centers must establish clear strategies for integrating AI-driven workflow changes to optimize efficiency while maintaining high standards of patient care. One key approach involves redesigning tasks to allow radiologists to focus on complex image interpretation and direct patient interactions while delegating routine or repetitive processes to automated AI tools [141]. However, for successful implementation, radiologists should play a central role in defining AI system performance parameters and must have the ability to pause or override the system if clinical targets are not met [142]. Ensuring transparency and control over AI-assisted decision-making will be essential for fostering trust and adoption among healthcare professionals.

Additionally, defining clear roles, responsibilities, and liabilities for clinicians involved in AI-integrated workflows is crucial for smooth and effective implementation [143]. AI adoption is expected to contribute to a rising clinical workload, making it imperative that AI-driven workflows align seamlessly with existing administrative and logistical processes [144]. In many cases, these supporting processes may also require AI-driven optimizations to prevent additional administrative burdens on radiologists and radiographers. A well-coordinated, symbiotic relationship between AI automation and hospital workflow management will be essential to maximizing the benefits of AI in radiology while ensuring operational efficiency and clinician satisfaction.

#### 4.3.4. Current Recommendations

The prominence of segmentation among the included studies is unsurprising, given that it represents one of the earliest applications of AI in medical imaging [145]. However, a key limitation in comparing these studies is the heterogeneity of computational metrics and the lack of standardization for clinical implementation. Additionally, over a third of the segmentation studies (10/26, 38.5%) did not report reasoning time alongside accuracy improvements, limiting the ability to assess their potential clinical applicability. Despite these limitations, common trends in convolutional neural network (CNN) usage were observed across the included segmentation studies. UNet, an encoder–decoder CNN with skip connections and a fully convolutional architecture, enables the preservation of spatial information and achieves high precision in pixel-level segmentation. As a result, it has become the mainstream choice for medical image segmentation [146].

The majority of the included segmentation studies employed UNet, either in customized forms or in combination with other CNNs for specific segmentation tasks. One study focusing on liver segmentation [102] utilized UNet++, an enhanced variant of UNet that incorporates denser skip connections between different stages to reduce the semantic gap between the encoder and decoder. Although this approach improved efficiency, the dice similarity coefficient (DSC) for liver tumor segmentation remained below 0.7, indicating room for further accuracy improvements. Two studies that demonstrated overall productivity gains [47,62] applied U-Net architectures with an EfficientNet encoder. EfficientNet encoders require up to eight times fewer parameters compared to similar networks, making them well-suited for building efficient models with limited computational resources [147]. Despite these resource optimizations, both studies maintained high segmentation accuracy, reporting strong DSC scores between 0.97 and 0.99. This suggests that the combination of U-Net architectures with EfficientNet encoders is an effective approach for achieving both accuracy and computational efficiency in medical image segmentation.

Among the included studies focusing on reduced reading times, the choice of AI model for lesion classification was largely determined by the anatomical region and disease pathology under investigation. ResNet is one of the most widely recommended deep learning models for classification due to its superior feature extraction capabilities [148]. It was employed in several studies [54,72] examining brain and spine pathologies, with one study identifying ResNet50 as the most effective pre-trained model for discriminating lesions in multiple sclerosis (MS), achieving an AUC of 0.81. However, the same study demonstrated even greater accuracy with a custom-designed network, achieving an AUC of 0.90. This aligns with a broader trend observed in other studies comparing CNN performances, where task-specific architectures often outperformed standard pre-trained models. Despite variations in model selection, all studies reported measurable improvements in classification accuracy with AI assistance. However, studies that failed to clarify productivity gains did not provide a comparison of AI processing times against human reading times, limiting their clinical applicability.

Rather than focusing solely on model selection, our analysis suggests that future research should prioritize simulated AI applications in time-constrained settings. These studies should incorporate a diverse range of readers with varying levels of experience, not just expert radiologists, to better reflect real-world clinical workflows. An example of this approach is seen in one of the included studies [51], which focused on automated classification of lumbar spine central canal and lateral recess stenosis. Conducted under standardized reading conditions, the study found that AI-assisted reporting required 47 to 71 s, compared to 124 to 274 s for unassisted radiologists. Additionally, DL-assisted general and in-training radiologists demonstrated improved interobserver agreement for four-class neural foraminal stenosis, with κ values of 0.71 and 0.70 (with DL) vs. 0.39 and 0.39 (without DL), respectively (both *p* < 0.001). Beyond the measurable gains in efficiency and accuracy, the study highlights the advantages of an “AI and human collaboration model”, which should be a focal point for future research.

Among all productivity themes analyzed, the use of AI for reducing MRI scanner time demonstrated the highest success in achieving measurable productivity gains, with 84.6% (11/13) of the included studies reporting positive outcomes. Even in studies where productivity losses were observed [55,76], improvements in image quality—such as enhanced signal-to-noise ratio (SNR) and contrast-to-noise ratio (CNR)—were still achieved. However, these studies did not clarify how such enhancements would translate to improved classification or diagnostic accuracy. Despite the demonstrated benefits, only a limited number of deep learning-based reconstruction (DLR) techniques are currently available for clinical MRI scanners [149]. Moreover, most of these techniques remain vendor-specific, including Advanced Intelligent Clear-IQ Engine (AiCE; Canon Medical Systems), AIR Recon DL (GE Healthcare), and Deep Resolve (Siemens). Another significant barrier to broader adoption is the lack of publicly available training datasets, which restricts independent advancements and customization for different clinical settings [150].

To overcome these challenges, our study recommends further vendor-agnostic research aimed at improving image quality, particularly for older MRI scanners from various manufacturers. One example is a multicenter study [69] that investigated deep learning reconstruction for brain MRI using SubtleMR, an FDA-cleared, vendor-agnostic CNN trained on thousands of MRI datasets spanning multiple vendors, scanner models, field strengths, and clinical sites. By leveraging such diverse datasets, studies can provide stronger evidence that deep learning-based processing enhances MRI efficiency and diagnostic value across different clinical environments.

Because of the limited number of studies demonstrating novel improvements in workflow processes or workload reduction, our paper advocates for increased integration of Transformer-based architectures and large language models (LLMs) in future machine learning (ML) applications for MRI. These advancements could facilitate automated draft report generation, standardization of clinical protocols, and triaging of complex cases by leveraging information from request forms and electronic medical records [151]. Given their low customization costs and compatibility with existing IT infrastructure [152], LLMs have the potential to surpass other AI tools in overcoming current technological barriers. Recent innovations, such as voice assistants, could further enhance the utility of LLMs by enabling conversational interactions. Integrating LLMs into existing speech recognition dictation systems used in radiology departments may offer real-time productivity benefits, fostering seamless collaboration between AI and human radiologists. This approach could help mitigate potential time losses or increased workload associated with text-based reporting [153].

Despite the early success of LLMs in other imaging modalities, such as radiograph interpretation [154], recent studies evaluating their applicability in MRI have highlighted several challenges. One major limitation is that human–LLM interactions have primarily been assessed in controlled environments rather than real-world clinical settings, where frequent interruptions and high workloads influence radiologists’ behavior [155]. Additionally, the phenomenon of “hallucinations” in generative AI remains a significant barrier to its adoption in MRI-reporting workflows [84]. Future LLM must address these shortcomings to ensure reliability, safety, and clinical trustworthiness. As with other emerging AI applications, the successful integration of LLMs in MRI will require close collaboration among AI developers, clinical radiologists, healthcare administrators, and regulatory bodies. Establishing best practices for ethical and efficient implementation will also be critical for ensuring the widespread adoption of LLMs across MRI and other medical specialties [156].

#### 4.3.5. Future Directions

Looking ahead, more standardized and multicenter prospective studies are needed to validate AI models under real-world conditions. Such trials could compare AI-driven MRI workflows with conventional practices, providing clearer insights into time savings, cost-effectiveness, and overall patient outcomes. In parallel, guidelines such as the recent PRISMA extensions for AI research [157] should continue to evolve in step with rapidly expanding AI research and applications. Establishing standardized productivity metrics, such as reduced turnaround times, minimized radiologist fatigue, and improved throughput would allow more meaningful comparisons across different studies.

Beyond technical measures, future research should also focus on user experiences, radiologist satisfaction, and patient-centered outcomes. While improvements in conventional metrics remain valuable, AI imaging has significant potential to reduce the fatigue and cognitive overload commonly experienced by radiologists. Burnout is a pressing concern, with one systematic review [158] finding rates as high as 88% in some regions. Although AI could theoretically mitigate burnout by streamlining tasks and maintaining diagnostic performance during prolonged reading sessions, a cross-sectional study [159] indicated that frequent AI use may paradoxically raise burnout risks if ease of adoption, radiologist satisfaction, and workflow integration are not properly addressed. Thus, tackling these interlinked factors is vital for the long-term viability of AI tools in MRI.

Lastly, initiatives that include iterative feedback loops, where radiologists refine AI outputs, could enhance model accuracy, yield institution-specific solutions, and build trust in AI recommendations. However, integrating AI into radiological workflows is not easy and requires significant resources and time, highlighting the need for strategies to continuously retrain and adapt models to evolving clinical settings [121]. Diversifying data sources, for instance by involving radiology residents in dataset creation [160], could lessen the load on senior radiologists and bolster model robustness. In addition, incorporating AI education into the radiology residency curriculum will equip future radiologists with the necessary technical competencies [161]. Further evidence is also required to support vendor-neutral platforms that can seamlessly integrate AI systems from multiple developers, thereby improving MRI access and facilitating enhanced combined reporting [162,163].

## 5. Conclusions

This review highlights significant advances in productivity metrics achieved through AI applications in MRI across various specialties, particularly in automating segmentation and reducing both scanning and reading times. Although many studies showed improvements in diagnostic accuracy, quantitative descriptions of efficiency gains were often limited. To enable meaningful clinical translation, future AI models must prioritize maintaining or improving productivity metrics without compromising diagnostic accuracy. In order to tackle underexplored themes in optimizing workflow and reducing workload, our study recommends increased focus and incorporation of LLM advancements in MRI workflow in order to accelerate radiologist engagement with substantial volumes of textual information encountered in day-to-day clinical practice. Good practices seen in other imaging modalities, where topics such as large-scale workload reduction or refined worklist triage have seen more maturity, should be explored and adopted in MRI [164,165].

Collaborative efforts among researchers, clinicians, and industry partners remain essential for developing standardized benchmarks and guidelines to measure productivity. Encouragingly, emerging initiatives are also addressing reproducibility concerns by integrating feedback loops for continuous model retraining and optimization. Exploring alternative training data sources could further reduce radiologist workload while retaining the credibility of reported productivity metrics. Ultimately, striking the right balance between efficacy, safety, and ease of implementation will be key to ensuring that AI-driven tools can meaningfully enhance radiologist productivity in MRI.

## Figures and Tables

**Table 1:** Summary of selected studies.

No.	Ref No.	Application and Primary Outcome Measure	Sample Size *	Region Studied	MRI Sequences Used	AI Technique Used	Key Results
1	[27]	Develop AI assistant to automatically segment breast tumors by capturing dynamic changes in multi-phase DCE-MRI with spatial-temporal framework	2190	Breast	DCE-MRI	UNet	DSC 92.6% internal + external testing set
2	[28]	Evaluate temporally and spatially resolved (4D) radiomics approach to distinguish benign from malignant enhancing breast lesions—avoiding unnecessary biopsies	329	Breast	DCE-MRI	Neural Network (PCA AI classifier)	AUC 80.6% (training dataset) + 83.5% (testing dataset)
3	[29]	Propose a SwinHR scheme for breast tumor segmentation on DCE-MRI, which integrates prior hemodynamic knowledge of DCE-MRI and Swin Transformer with RLK blocks to extract temporal and spatial information	2246	Breast	DCE-MRI	CNN	Dice 0.75–0.81 across different datasets
4	[30]	Develop image-based automatic deep learning method to classify CMR images by sequence type and imaging plane for improved clinical post-processing efficiency	334	Chest (Cardiac)	Cine-CMR, T1w/T2w/T2*w, Perfusion, LGE	CNN	Accuracy and F1-scores = 81.8% and 0.82 for SVT, 94.3% and 0.94 MVT on the hold-out test set
5	[31]	Evaluating AI tool in interpreting MR imaging to produce aortic root and valve measurements by comparing accuracy and efficiency with cardiologists	35	Chest (Cardiac)	Cine-CMR	CNN	ICC = 0.98 between AI assessment of aortic root and valve with cardiologists
6	[32]	Accelerate multiple b-value gas DW-MRI for lung morphometry using deep learning	101	Chest (Lungs)	DW-MRI	DC -RDN	Improved reconstruction acceleration factor of 4
7	[33]	Develop and test a comprehensive deep learning-based segmentation system to improve automatic upper airway segmentation in static and dynamic MRI	160	Chest (Lungs)	Static/dynamic 2D + 3D MR	UNet	DSC 0.84 to 0.89 across different sequences
8	[34]	Implement a deep-learning approach for segmentation of liver tumors in magnetic resonance imaging using UNet++	105	Abdomen (Liver)	T1w arterial phase, T2w	UNet ++	DSC liver and tumor segmentations > 0.9 and >0.6 respectively
9	[35]	Develop and evaluate a DCNN for automated liver segmentation, volumetry, and radiomic feature extraction on contrast-enhanced portal venous MRI	470	Abdomen (Liver)	3D T1w portal venous	DCNN	DSC internal + external + public testing set 0.93–0.97
10	[36]	Train deep learning model to differentiate pathologically proven HCC and non-HCC lesions including lesions with atypical imaging features on MRI.	118	Abdomen (Liver)	Multi-phasic T1w	CNN	Sensitivities/specificities for HCC + non-HCC lesions = 92.7%/82.0% and 82.0%/92.7%; AOC = 0.912
11	[37]	Explore the feasibility of deep learning (DL) features derived from gadoxetate disodium (Gd-EOB-DTPA) MRI, qualitative features, and clinical variables for predicting early recurrence.	285	Abdomen (Liver)	Contrast enhanced MRI	VGGNet-19	DL vs. clinical nomogram AUC 0.91–0.95 vs. 0.72–0.75 for training and validation set
12	[38]	Investigate the utility of DL automated segmentation-based MRI radiomic features and clinical-radiological characteristics in predicting early recurrence after curative resection of single HCC	434	Abdomen (Liver)	T2w	UNet	AUC 0.743 vs. 0.55–0.64 in widely adopted BCLC and CNLC systems
13	[39]	Develop and validate a ML mortality risk quantification method for HCC patients using clinical data and liver radiomics on baseline MRI	555	Abdomen (Liver)	T1w breath-hold sequences before + after contrast	CNN	C-indices of 0.8503 and 0.8234
14	[40]	Develop and evaluate a CNN based algorithm to evaluate hepatobiliary phase (HBP) adequacy of gadoxetate disodium (EOB)-enhanced MRI and reduce examination time	484	Abdomen (Liver)	EOB-enhanced MRI-HBP	CNN	Kappa 0.67–0.80 and AUC = 0.95–0.97 for internal and external test set
15	[41]	Develop AI model for prostate segmentation and PCa detection, and explore CAD compared to conventional PI-RADS assessment	100	Abdomen (Prostate)	Not specified	CNN	Prostate mean DSCs fine + coarse segmentation = 0.91 + 0.88. PCa diagnosis AI CAD > consistency in internal (kappa 1.00) + external (kappa 0.96) tests vs. radiologists (kappa 0.75)
16	[42]	Utilize a DL AI workflow for automated EPE grading from prostate T2W MRI, ADC map, and High B DWI.	634	Abdomen (Prostate)	T2W MRI, ADC map, and High B DWI	CNN	Sensitivity of 0.67, specificity of 0.73, accuracy of 0.72
17	[43]	Study the effect of artificial intelligence (AI) on the diagnostic performance of radiologists in interpreting prostate mpMRI images of the PI-RADS category 3.	87	Abdomen (Prostate)	mpMRI	CNN	AI with radiologists => diagnostic specificity + accuracy (0.695 vs. 0.000 and 0.736 vs. 0.322, *p* < 0.001)
18	[44]	Assess PI-RADS-trained DL algorithm performance and investigate the effect of data size and prior knowledge on the detection of csPCa in biopsy-naïve men with a suspicion of PCa.	2734	Abdomen (Prostate)	mpMRI	UNet	DL sensitivity for detecting PI-RADS ≥ 4 lesions = 87%; AUC of 0.88.
19	[45]	To evaluate whether an AI-driven algorithm can detect clinically significant PCa (csPCa) in patients under AS	56	Abdomen (Prostate)	mpMRI	CNN	Patient sensitivity/specificity = 92.5%/31% detection of ISUP ≥ 1 and 96.4%/25% detection of ISUP ≥ 2
20	[46]	Investigate image quality, efficiency, and diagnostic performance of a DLSB against BLADE for T2WI for GC	112	Abdomen (Stomach)	DLSB - T2w	CNN	W test 0.679–0.869 subjective IQS; ICC 0.597–0.670 measure SNR and CNR; Kappa 0.683–0.703 agreement cT stage
21	[47]	Develop, validate, and deploy deep learning for automated TKV measurement on T2WI MRI studies of ADPKD	129	Abdomen (Kidney)	T2w	UNet, EfficientNet	DSC > 0.97 + Bland–Altman mean percentage difference < 3.6%.
22	[48]	Analyzes potential cost-effectiveness of integrating an AI–assisted system into differentiation of incidental renal lesions as benign or malignant on MRI follow-up	NA	Abdomen (Kidney)	T2w and T1 post contrast	ResNet	8.76 and 8.77 QALYs for MRI vs. MRI + AI strategy
23	[49]	Develop a DL technique for fully automated segmentation of multiple OARs on clinical abdominal MRI with high accuracy, reliability, and efficiency	102	Abdomen	T1-VIBE	U-Net	DSC 0.87–0.96
24	[50]	Develop a DL model for automated detection and classification of lumbar central canal, lateral recess, and neural foraminal stenosis	446	Spine	Axial T2w, Sagittal T1w	CNN	Internal testing classification = kappa 0.89–0.95. External testing classification = kappa 0.95–0.96
25	[51]	To assess the speed and interobserver agreement of radiologists for reporting lumbar spinal stenosis with and without DL assistance	25	Spine	Axial T2w, sagittal T1w	CNN	Kappa 0.71 + 0.70 (DL) vs. 0.39 + 0.39 (no DL) for DL-assisted general + in-training radiologists (*p* < 0.001)
26	[52]	To externally validate SpineNet predictions for disk degeneration (DD) using Pfirrmann classification and Modic changes (MCs)	1331	Spine	T2w	SpineNet (DCNN)	DD and MC accuracy = 78% and 86%. PG = Lin concordance correlation coefficient 0.86 and kappa 0.68
27	[53]	To evaluate SpineNet (SN) ratings compared with those of an expert radiologist in analysis of degenerative features in MRI scans	882	Spine	Not specified	SpineNet (DCNN)	Kappa 0.63–0.77 for PG, kappa 0.07–60 for SL, kappa 0.17–0.57 for CCS
28	[54]	Evaluate a deep learning model for automated and interpretable classification of central canal stenosis, neural foraminal stenosis, and facet arthropathy from lumbar spine MRI	200	Spine	T2WI axial	V-Net, ResNet-50	Dural sac + intervertebral disk = DSC 0.93 and 0.94. Localization foramen and facet = 0.72 and 0.83. CCS = kappa 0.54. Neural foraminal stenosis and facet arthropathy = AUC 0.92 and 0.93
29	[55]	Develop and validate an AI-based model that automatically classifies lumbar central canal stenosis (LCCS) using sagittal T2w MRIs	186	Spine	Sagittal T2w MRI	CNN	Multiclass model = kappa 0.86 vs. 0.73–0.85 for readers. Binary model = AUC 0.98; sensitivity 93% + specificity 91% vs. 98–99% and 54–74% for readers
30	[56]	To compare the image quality and diagnostic performance between standard TSE MRI and accelerated MRI with deep learning (DL)-based image reconstruction for degenerative lumbar spine diseases	50	Spine	T1WI, T2WI	CNN	DL_coarse + DL_fine = significantly higher SNRs T1WI and T2WI, higher CNRs on T1WI, similar CNRs on T2WI
31	[57]	To develop a DL model to accurately detect ACL ruptures on MRI and evaluate effect on accuracy and efficiency of clinicians	3800 MRIs	MSK	Not specified	CNN	AUC 0.987, sensitivity + specificity 95.1%
32	[58]	To analyze occupation ratio using a DL framework and study fatty infiltration of supraspinatus muscle using automated region-based Otsu thresholding technique for rotator cuff tears	240	MSK	T1WI fast spin echo	CNN	Mean DSC/accuracy/sensitivity/specificity/relative area difference segmented lesion = 0.97/99.84/96.89/99.92/0.07
33	[59]	To establish an automated, multitask, MRI-based deep learning system for the detailed evaluation of supraspinatus tendon (SST) injuries	3087	MSK	Not specified	VGG16	AUC of VGG16 = 0.92, 0.97–0.99 for RC-MTL classifiers. ICCs of the radiologists = 0.97–0.99
34	[60]	Evaluate performance of automated reconstruction algorithm to reconstruct denoised images from undersampled MR images in patients with shoulder pain	38	MSK	TSE fat saturation, sagittal T2w TSE, coronal T1w TSE	CNN	Kappa 0.95; Improved SNR and CNR (*p* < 0.04)
35	[61]	Investigate feasibility of DL-MRI in shoulder imaging	400	MSK	PD, T2w	CNN	DL-MRI (Kendall W: 0.588~0.902); non-DL-MRI (Kendall W: 0751~0.865)
36	[62]	DL assessment of lower extremities in patients with lipedema or lymphedema. Develop pipeline for landmark detection methods and segmentation	45	MSK	3D DIXON MR lymphangiography	EfficientNet-B1 UNet	Z-deviation (landmark) = 4.5 mm and segmentation DSC = 0.989
37	[63]	To propose a deep neural network for diagnosis of MR Images of tumors of the hand in order to better define preoperative diagnosis and standardize surgical treatment	221	MSK	T1w axial/sagittal/coronal	DeepLabv3+ DCNN, TensorFlow	Average confidence level of 71.6% in segmentation of hand tumors
38	[64]	Develop an Anatomical Context-Encoding Network (ACEnet) to incorporate 3D spatial and anatomical contexts in 2D CNNs for efficient and accurate segmentation of brain structures from MRI	30	Brain	T1w	UNet	Skull stripping DSC > 0.976
39	[65]	To verify reliability of volumes automatically segmented using AI-based application and evaluate changes in brain and CSF volume with healthy aging	133	Brain	3D T1w	UNet	Mean ICC 0.986; 95% CI, 0.981–0.990
40	[66]	To evaluate inference times of novel PSFNet that synergistically fuses SS (scan specific) and SG (scan general) priors for performant MRI reconstruction in low-data regimes	NA	Brain	Not specified	PSFNet	3.1 dB higher PSNR, 2.8% higher SSIM, and 0.3 × lower RMSE vs. baselines
41	[67]	Rapid estimation of multiparametric T1, T2, PD, 3D-QALAS measurements using SSL	NA	Brain	T1w, T2w, PD, and inversion efficiency maps	SSL-based QALAS	RMSE dictionary matching and SSL-QALAS methods = 8.62, 8.75, 9.98, and 2.42% for T1, T2, PD, and IE sequences
42	[68]	Compare effect of head motion-induced artifacts on the consistency of MR segmentation performed by DL and non-DL methods	110	Brain	T1w	ReSeg, FastSurfer, Kwyk	DSC 0.91–0.96 for DL vs. 0.88–0.91 non-DL
43	[69]	Evaluate and compare both image quality and quantitative image-analysis consistency of 60% accelerated volumetric MR imaging sequences with SubtleMR with standard of care (SOC)	40	Brain	3D T1w	SubtleMR CNN	Both FAST-DL and SOC statistically superior to FAST for all analyzed features (*p* < 0.001)
44	[70]	To demonstrate a 3D CNN that performs expert-level, automated meningioma segmentation and volume estimation on MRI	10,099	Brain	T1w	UNet	Tumor segmentation DSC = 85.2% vs. experts 80.0–90.3%
45	[71]	Examine efficacy of new approach to classification of brain tumor MRIs through VGG19 features extractor and PGGAN augmentation model	233	Brain	T1w CE	VGG19 + PGGAN	VGG19 + model multiplied dataset ~ 6-fold + 96.59% accuracy. PGGAN model > accuracy VGG19 + model by >1.96%
46	[72]	Investigate the potential of DL to differentiate active and inactive lesions in MS using non-contrast FLAIR-type MRI data	130	Brain	T1w FLAIR	Multiple CNNs	Accuracy 85%, sensitivity 95%, specificity 75%, AUC = 0.90. ResNet50 most effective
47	[73]	To propose a DL framework that combines multi-scale attention guidance and multi-branch feature processing modules to diagnose PD by learning sMRI T2w	504	Brain	T2w	DCNN	Accuracy 92%, sensitivity 94%, specificity 90% F1 score 95% for identifying PD and HC
48	[74]	To propose a fully automatic pipeline for measuring the magnetic resonance parkinsonism index (MRPI) using deep learning methods	400	Brain	T1w	nnUNet, HRNet	Inter-rater APE on external datasets = 11.31%
49	[75]	Automatically diagnose PD from HCs with segmentation using VB-net and radiomics brain features	246	Brain	T1w	3D VB-net	AUCs 0.988 and 0.976 in training and testing set
50	[76]	Evaluate whether DLR improves the image quality of intracranial MRA at 1.5 T	40	Brain	Axial and coronal MIP images	AI Clear IQ Engine—DLR	SNR and CNR for basilar artery > in DLR images (*p* < 0.001)
51	[77]	Develop and validate intelligent hypoxic–ischemic encephalopathy (HIE) identification model	186 (HIE) + 219 (HC)	Brain	NA	DLCRN	AUC 0.868/0.813/0.798 for training/internal/independent cohorts
52	[78]	DL model to predict 2-year neurodevelopmental outcomes in neonates with HIE using MRI	414	Brain	T1w, T2w, and diffusion tensor imaging	OPiNE (CNN)	AUC 0.74 and 63% accuracy in-distribution test set + AUC 0.77 and 78% accuracy out-of-distribution test set
53	[79]	Automatic segmentation-based method for 2D biometric measurements fetal brain on MRI	268	Brain	T2w SSFSE, (BSSFP), T1w and DWI	nnUNet	Correlation coefficients CBPD, TCD, LAD and RAD = 0.977, 0.990, 0.817, 0.719
54	[80]	LVPA-UNet model based on 2D-3D architecture to improve GTV segmentation for radiotherapy in nasopharyngeal carcinoma	1010	ENT	T1w	LVPA-UNet	>DSC by 2.14%, precision by 2.96%, recall by 1.01%, reduction HD95 0.543 mm
55	[81]	Evaluate DL-CAD system on radiologists’ interpretation accuracy and efficiency in reading bi-parametric prostate magnetic resonance imaging scans	100	Abdomen (Prostate)	Not specified	DL-CAD	DL-CAD vs. radiologist = AUC 0.88 vs. 0.84
56	[82]	Determine if AI systems for brain MRI could be used as a clinical decision support tool to augment radiologist performance	390	Brain	T1w, FLAIR, GRE, T1-post diffusion	CNNs + Bayesian network	Radiology resident performance > with ARIES for = TDx (55% vs. 30%; *p* < 0.001) and T3DDx (79% vs. 52%; *p* = 0.002)
57	[83]	To evaluate the learning progress of less experienced readers in prostate MRI segmentation—in preparation for datasets in AI development	100 MRIs	Abdomen (Prostate)	T2w, DWI	RECOMIA	Using DSC > 0.8 as threshold, residents = 99.2%; novices = 47.5%/68.3%/84.0% with subsequent rounds of teaching on accurate segmentations
58	[84]	Efficacy of AI-generated radiology reports and contribution to the advancement of radiology workflow	685 MRI reports	NA	NA	GPT-3.5-turbo	High scores comprehension (2.71 ± 0.73) + patient-friendliness (4.69 ± 0.48) (*p* < 0.001). 1.12% hallucinations and 7.40% potentially harmful translations
59	[85]	Evaluate intelligent automation software (Jazz) in assessing new, slowly expanding, and contrast-enhancing MS lesions	117 MRIs	Neurology	T1w	Jazz	Kappa 0.5
60	[86]	Evaluate protocol determination with CNN classifier based on short-text classification and comparing protocols determined by MSK radiologists	6275 MRIs	MSK	NA	ConvNet	CNN and radiologist = Kappa 0.88. Routine protocols and tumor protocols = AUC 0.977
61	[22]	Efficient approach for segmentation relevant for diagnosis and treatment of OSAS	181	ENT	T1w, T2w TSE	UNet	DSC (test set) = 0.89, 0.87, 0.79 (tongue, pharynx, soft palate)
62	[87]	Develop an efficient preoperative MRI radiomics evaluation approach of ALN status	1088	Breast	T2w, DWI-ADC, T1 + contrast	ML random forest algorithm	ALN-tumor radiomic signature AUC = 0.87–0.88 in training, external, prospective-retrospective validation cohort
63	[20]	Investigate automatically identifying normal scans in ultrafast breast MRI with AI to increase efficiency and reduce workload	438	Breast	TWIST sequences	ResNet-34	AUC 0.81
64	[88]	Framework for reconstructing dynamic sequences of 2D CMR from undersampled data using CNNs	5	Chest (Cardiac)	2D CMR	CNN	Preserves anatomical structures up to 11-fold under sampling
65	[89]	Fast and high-quality reconstruction of clinical accelerated multi-coil MR data by learning a VN using DL	50	MSK	2D TSE	CNN	VN reconstructions > standard algorithms and acceleration factor 4
66	[90]	Develop DL based automated OAR delineation method achieving reliable expert performance	70	Head and Neck	T1w	UNet	Average DSC 0.86 (0.73–0.97)—6% better than competing methods

Not available (NA), Artificial intelligence (AI), magnetic resonance imaging (MRI), T1-weighted (T1w), T2-weighted (T2w), T2*-weighted (T2*w), area under the curve (AUC), convolutional neural network (CNN), deep learning (DL), deep convolutional neural network (DCNN), deep cascade of residual dense network (DC-RDN), principal component analysis (PCA), diffusion-weighted imaging (DWI), dice similarity coefficient (DSC), dynamic contrast-enhanced (DCE), cardiac magnetic resonance (CMR), late gadolinium enhancement (LGE), root mean squared error (RMSE), peak signal to noise ratio (PSNR), Structural similarity (SSIM), healthy control (HC), hepatocellular carcinoma (HCC), prostate imaging-reporting and data system (PI-RADs), intraclass and concordance correlation coefficients (ICC), prostate cancer (PCa), self-supervised learning (SSL), computer-aided diagnosis (CAD), proton density (PD), multiparametric MRI (mpMRI), International Society of Urological Pathology (ISUP), turbo spin echo (TSE), ear nose throat (ENT), spondylolisthesis (SL), Pfirrmann grading (PG), central canal stenosis (CCS), anterior cruciate ligament (ACL), musculoskeletal (MSK), adaptive radiology interpretation and education system (ARIES). * Numbers are patients, unless stated otherwise.

**Table 2:** Productivity measures of selected studies.

No.	Relevant Study Finding(s)	Study Center Size	Reduced Scan Time	Automated Segmentation	Optimized Worklist Triage and Workflow	Time Savings and Workload Reduction	Decreased Reading Time	Overall Productivity Gain
1	Time for manual annotation reduced by factor of 20, while maintaining physician accuracy.	M	No	Yes	No	No	No	Gain
2	Fewer unnecessary biopsies of benign breast lesions by ~36.2%, *p* < 0.001 at the cost of up to 4.5% (n = 4) false negative low-risk cancers	S	No	Yes	No	No	No	Unclear—no dedicated reproducibility analysis of the automated lesion segmentation and feature extraction
3	Experimental evidence showed superiority to other available automated segmentation methods	M	No	Yes	No	No	No	Unclear—ablation studies used but no reasoning time or comparison to manual methods
4	Fully automated multivendor model that classifies cardiac MR images directly from scanner for post processing, validated on external data with high accuracy	M	No	No	Yes	No	No	Unclear—no reasoning time and only trained to recognize standard cardiac anatomy
5	AI delivered measurements in 2.6 s vs. mean of 334.5 s by cardiologists, without compromising accuracy.	S	No	No	No	Yes	No	Gain
6	Breath-holding reduced 17.8 s to 4.7 s; slice reconstruction within 7.2 ms	S	Yes	No	No	No	No	Gain
7	Segmentation time per subject/per slice for static 3D and dynamic 2D MRI less than 1.2 and 0.01 s respectively	S	No	Yes	No	No	No	Gain
8	Automated segmentation of liver and liver tumor saved >160 and >20 s, respectively, on average per case	S	No	Yes	No	No	No	Unclear—Despite time gains, DSC liver tumor segmentation < 0.7, raising concerns with accuracy
9	Total training time 6.35 h. Diverse training dataset of different hepatic etiologies. Mean run time of 0.52 s.	M	No	Yes	No	No	No	Gain
10	CNN accuracy of 87.3% and computational time of <3 ms	S	No	No	Yes	No	No	Gain
11	Proposed DL nomogram superior to clinical nomogram in predicting early recurrence for HCC patients post hepatectomy	M	No	No	Yes	No	No	Unclear—no reasoning time provided
12	DL-assisted automated segmentation using radiomic signatures allows better prediction of early HCC recurrence	S	No	Yes	No	No	No	Unclear—no reasoning time provided
13	1.11 min risk prediction framework on average	S	No	No	Yes	No	No	Gain
14	48% examinations achieved <20 min HPB delay normally applied	M	Yes	No	No	No	No	Gain
15	AI-first read (8.54 s) was faster than readers (92.72 s) and the concurrent-read method (29.15 s)	M	No	Yes	No	No	Yes	Gain
16	Improved accuracy compared against expert genitourinary radiologist	S	No	No	Yes	No	No	Unclear—no reasoning time; only compared with the performance of one expert radiologist
17	AI reduced reading time (mean: 351 s, *p* < 0.001)	M	No	No	No	No	Yes	Gain
18	DL sensitivity for the detection of Gleason > 6 lesions was 85% compared to 91% for a consensus panel of expert radiologists	M	No	No	No	No	Yes	Loss—no improvement in reasoning time shown, poor accuracy vs. expert unless high training case count met
19	Accurate automated AI-algorithm for prostate lesion detection and classification	S	No	No	Yes	No	No	Unclear—no reasoning time provided; adopting to clinical practice unclear
20	Vs. BLADE, DLSB reduced acquisition time of T2WI from 4 min 42 s/patient to 18 s/patient, with better overall image and comparable accuracy	S	Yes	No	No	No	No	Gain
21	51% reduction in radiologist time for model-assisted segmentation	S	No	Yes	No	No	No	Gain
22	Additional use of AI-based algorithm may be cost-effective alternative in differentiation of incidental renal lesions on MRI	NA	No	Yes	No	No	No	Unclear—integration into clinical practice not clarified
23	Inference completed within 1 min for a three-dimensional volume of 320 × 288 × 180	S	No	Yes	No	No	No	Gain
24	DL model detecting and classifying lumbar spinal stenosis at MRI comparable with radiologists for central canal and lateral recess stenosis	S	No	No	No	No	Yes	Unclear—improvements on interpretation time not discussed
25	DL-assisted radiologists = reduced interpretation time per spine MRI study. Mean of 124–274 s reduced to 47–71 s (*p* < 0.001)	S	No	No	No	No	Yes	Gain
26	20.83% of disks rated differently by SpineNet compared to human raters, but only 0.85% of disks had a grade difference > 1	S	No	No	No	No	Yes	Unclear—improvements on interpretation time not discussed
27	SpineNet recorded > pathological features in PG vs. radiologist	S	No	No	No	No	Yes	Unclear—improvements on interpretation time not discussed
28	Accurate automated grading lumbar spinal stenosis and facet arthropathy using axial sequences only	S	No	No	No	No	Yes	Unclear—improvements on interpretation time not discussed
29	Accurate AI-based lumbar central canal stenosis classification on sagittal MR images	S	No	No	No	No	Yes	Unclear—improvements on interpretation time not discussed, no external test set
30	Accelerated protocol reduced MRI acquisition time by 32.3% compared to standard protocol	S	Yes	No	No	No	No	Gain
31	AI assistance significantly improved accuracy (>96%) with reduction in diagnostic time observed (up to 9.7 s in expert or 24.5 s in trainee groups)	M	No	No	No	No	Yes	Gain
32	Analysis using freeware computer program that analyzed muscle atrophy and fatty infiltration MR images in <than 1 s	S	No	Yes	No	No	No	Gain
33	Automated multitask DL system in diagnosing SST injuries with comparable accuracy to experts	S	No	No	No	No	Yes	Unclear—improvements on interpretation time not discussed
34	Reconstruction performed on the scanner in ~80 s for CS DL reconstructions compared to approximately ~40 s for standard CS reconstructions	S	Yes	No	No	No	No	Loss
35	Scan time of DL-MRI (6 min 1 s) ~50% decreased compared with that of non-DL-MRI (11 min 25 s)	S	Yes	No	No	No	No	Gain
36	Prediction time/patient for first + second landmark detection = 2.7 s + 0.1 s. Mean prediction time/patient for tissue segmentation 8 s	S	No	Yes	No	No	No	Gain
37	Automated segmentation of hand tumors with 71.6% accuracy	S	No	Yes	No	No	No	Unclear—poor accuracy, improvements on segmentation time not discussed
38	Slight computation cost of 16.6% increase from baseline; MRI head scan segmentation within ~9 s	S	No	Yes	No	No	No	Gain
39	Brain and intracranial CSF volume ratios measured within 1 min	S	No	Yes	No	No	No	Gain
40	PSFNet required order of magnitude lower samples and faster inference vs. SG methods	S	Yes	No	No	No	No	Gain
41	Reconstruction multiparametric maps using pre-trained SSL-QALAS model < 10 s, no external dictionary required. Scan-specific tuning with target subject < 15 min	S	Yes	No	No	No	No	Gain
42	ReSeg DCNN inference time 11 s with >consistent segmentations	S	No	Yes	No	No	No	Gain
43	60% reduction sequence scan time using FAST-DL—from 4 to 6 min 56 s to 2 min 40–44 s, with >quality	S	Yes	No	No	No	No	Gain
44	Reduced segmentation time by 99% (2 s/segmentation *p* < 0.001) + more accurate	S	No	Yes	No	No	No	Gain
45	Accurate DL model helping radiologists classify brain tumor types on MRI with less training data needed	S	No	No	No	Yes	No	Unclear—training time not compared, limited brain tumor types, inconsistent image sizes due to computational limits
46	Multiple CNNs allow accurate discerning active vs. inactive lesions without reliance on contrast agents on FLAIR images	M	No	No	No	No	Yes	Unclear—improvements on interpretation time not discussed
47	DL model can accurately distinguish PD and HC using T2w MRI	S	No	No	No	No	Yes	Unclear—improvements on interpretation time not discussed
48	Automated DL pipeline can accurately predict MRPI comparable with manual measurements	S	No	Yes	No	No	No	Unclear—limited training data, segmentation time not discussed
49	Automated segmentation of brain into 109 regions with distinguishing PD from HC in 1 min	S	No	No	No	No	Yes	Gain
50	DLR = higher quality 1.5 T intracranial MRA images with improved visualization of arteries	S	Yes	No	No	No	No	Loss—multifold increase in imaging time, benefits high image quality on disease detectability not detailed
51	DLCRN model allowing accurate identification of HIE in neonates using MRI	S	No	Yes	No	No	No	Unclear—improvement on segmentation time and clinical application not detailed
52	DL analysis neonatal brain MRI yielded high performance for predicting 2-year neurodevelopmental outcomes	M	No	Yes	No	No	No	Unclear—segmentation times and potential real time clinical application not discussed
53	Accurate deep segmentation network using 2D biometric measurements of fetal brain on MRI	S	No	Yes	No	No	No	Unclear—only normal fetal brains in training data, segmentation time vs. manual methods not detailed
54	Accurate GTV segmentation using T1w MRI for radiotherapy in nasopharyngeal carcinoma	M	No	Yes	No	No	No	Unclear—segmentation time not discussed
55	Median reading time reduced by 21% from 103 to 81 s (*p* < 0.001)	M	No	No	No	No	Yes	Gain
56	Improved diagnostic accuracy of radiology resident using ARIES support tool	S	No	No	No	Yes	No	Unclear—time gains or losses with support tool not discussed
57	Segmentation time of experienced resident 7 min 22 s—7 min 50 s per case	S	No	No	No	Yes	No	Gain
58	Good efficacy AI-generated radiology in terms of report summary accuracy and patient-friendliness	S	No	No	No	Yes	No	Unclear—artificial hallucinations and harmful translations might equate to more work to verify reports
59	Significantly more MS lesions detected vs. baseline. Reading time 2 min 33 s/case.	M	No	No	No	No	Yes	Gain
60	Training time approximately 10 min	S	No	No	Yes	No	No	Gain
61	Consistent with expert readings. Processing one dataset = ~2 s using modern GPU	S	No	Yes	No	No	No	Gain
62	Accurate signature that could be preoperatively used for identifying patients with ALN metastasis in early-stage invasive breast cancer	M	No	No	No	Yes	No	Unclear—potential time gains or losses with clinical application not discussed
63	Workload and scanning time reductions = 15.7% and 16.6%	S	Yes	No	No	Yes	No	Gain
64	Reconstruction < 10 s with each complete dynamic sequence and <23 ms for 2D case	S	Yes	No	No	No	No	Gain
65	Reconstruction time of 193 ms on single graphics card	S	Yes	No	No	No	No	Gain
66	Accurate DL framework for automated delineation of OARs in HN radiotherapy treatment planning	S	No	Yes	No	No	No	Unclear—segmentation time not discussed

Single center study (S), multicenter study (M), hepatocellular carcinoma (HCC).

## Data Availability

Not applicable.
